# Coronavirus M protein impairs cilium during early infection by enhancing the AurA-HDAC6 axis

**DOI:** 10.1371/journal.ppat.1013515

**Published:** 2025-09-12

**Authors:** Tenghan Zhuang, Peng Yang, Mengqin Wang, Shiyu Liu, Wei Wang, Boyan Sun, Yue Xu, Li Chen, Xi Bao, Danchen Aaron Yang, Yongqian Zhao, Baochao Fan, Lei Feng, Bin Li

**Affiliations:** 1 Institute of Veterinary Immunology and Engineering, National Research Center of Engineering and Technology for Veterinary Biologicals, Jiangsu Academy of Agricultural Sciences, Nanjing, Jiangsu, China; 2 GuoTai (Taizhou) Center of Technology Innovation for Veterinary Biologicals, Taizhou, Jiangsu, China; 3 Jiangsu Key Laboratory for Food Quality and Safety-State Key Laboratory Cultivation Base of Ministry of Science and Technology, Nanjing, Jiangsu, China; 4 College of Veterinary Medicine, Nanjing Agricultural University, Nanjing, Jiangsu, China; 5 Institute of Veterinary Medicine, Key Laboratory of Veterinary Biological Engineering and Technology, Jiangsu Academy of Agricultural Sciences, Nanjing, Jiangsu, China; 6 School of Pharmacy, Jiangsu University, Zhenjiang, Jiangsu, China; University of Maryland School of Medicine, UNITED STATES OF AMERICA

## Abstract

Coronaviruses (CoVs) are implicated in human outbreaks and significant economic losses in the porcine and avian industries. Recent investigations have underscored the potential role of cilia within the respiratory tracts of infected hosts, particularly regarding the entry of severe acute respiratory syndrome coronavirus 2 (SARS-CoV-2). However, the mechanisms by which other CoVs exert their virulence through ciliary interactions remain inadequately elucidated. In this context, our research has demonstrated that porcine epidemic diarrhea virus (PEDV) and porcine deltacoronavirus (PDCoV) induce ciliary disassembly within six hours post-infection during the early infection stage. Utilizing mass spectrometry, we identified histone deacetylases 6 (HDAC6) or Aurora A (AurA) as binding partners of PEDV or PDCoV membrane (M) proteins. Immunofluorescence studies corroborated that the AurA-HDAC6 axis serves as a principal regulator of ciliary disassembly. Additionally, M proteins from all four CoV genera—PEDV, SARS-CoV-2, PDCoV, and infectious bronchitis virus (IBV)—were observed to congregate at the ciliary base. Molecular techniques, including immunoprecipitation and molecular docking combined with molecular mechanics/generalized born surface area (MM/GBSA) free energy decomposition analysis, further revealed that CoV M proteins interact with both AurA and HDAC6. These interactions depend on conserved residues at the transmembrane-cytosolic junction of M proteins, essential for their binding to the AurA-HDAC6 axis. Mutations disrupting these residues significantly impaired the binding affinity, thus inhibiting the associated ciliary disassembly process. Collectively, our findings illuminate a conserved regulatory mechanism involving CoV M proteins across all four genera, contributing to ciliary disassembly during early infection. This work enhances our understanding of the fundamental interactions between CoVs and host cells, positioning AurA and HDAC6 as potential therapeutic targets for a broad spectrum of CoV infections.

## Author summary

CoVs represent a diverse group of pathogens that elicit infections in both humans and animals, with severe strains such as SARS-CoV-2 being responsible for the COVID-19 pandemic. While significant progress has been achieved in the treatment of COVID-19, there remains an urgent requirement for effective antiviral agents capable of providing broad-spectrum coverage against various CoVs. A comprehensive understanding of the interactions between CoVs and host cilia is essential for the identification of therapeutic and preventative targets. In the present study, we examined the role of the AurA-HDAC6 axis, which is implicated in the disassembly of cilia, during infections with porcine CoVs. Our results demonstrate that the M proteins across all four genera of CoVs interact with both AurA and HDAC6. Notably, mutations within the conserved amino acid sequences of the M proteins result in a diminished binding affinity for AurA and HDAC6, consequently impairing the capacity to induce ciliary disassembly. These findings underscore the significance of elucidating the mechanisms by which CoVs influence cilia and interact with AurA and HDAC6, thereby offering new insights into virus-host interactions and identifying potential therapeutic targets for antiviral interventions.

## Introduction

Coronaviruses (CoVs) are enveloped, positive-sense, single-stranded RNA viruses classified within the family *Coronaviridae*. This family is further divided into four genera: *alphacoronavirus*, *betacoronavirus*, *gammacoronavirus*, and *deltacoronavirus*. Infections caused by CoVs primarily lead to respiratory and enteric diseases [1]. The severe acute respiratory syndrome coronavirus 2 (SARS-CoV-2), which falls under the *betacoronavirus* genus, is the pathogen responsible for the COVID-19 pandemic [2]. Similarly, the infectious bronchitis virus (IBV), which belongs to the *gammacoronavirus* genus, primarily invades through the respiratory mucosa [3–5]. The porcine epidemic diarrhea virus (PEDV), an *alphacoronavirus,* and porcine deltacoronavirus (PDCoV) produce similar symptoms, including watery diarrhea, vomiting, dehydration, and a high mortality rate in neonatal piglets [6–8]. Mature virions of CoVs consist of four structural proteins: envelope (E), membrane (M), nucleocapsid (N), and spike (S) proteins [1,4].

The cilium is an antenna-like, membrane-bound organelle that protrudes from the apical surface of nearly all eukaryotic cells [9–11] and is dynamically assembled at the end of mitosis and disassembled during the G1-S transition and before mitotic entry [12,13]. The length of the cilium is contingent upon the balance between assembly and disassembly processes in coordination with the cell cycle [13,14]. The M [15] and N [16] proteins of PEDV have been reported to extend the S phase of the cell cycle, potentially leading to ciliary disassembly during the G1-S transition. Infection with SARS-CoV-2 results in a ciliary loss [17,18] and dedifferentiation of multiciliated cells [19], while infection with IBV leads to loss of ciliary movement [20]. However, whether CoV from all genera exerts virulence by causing ciliary damage remains to be determined.

Histone deacetylases (HDACs) comprise a family of enzymes tasked with deacetylating lysine residues [21]. This enzymatic activity is critical for various biological functions [22], including ciliary disassembly [23], viral pathogenesis [24,25], and tumorigenesis [26]. Among them, HDAC6 is unique as it predominantly resides in the cytoplasm, featuring two catalytic domains and a ubiquitin-binding domain. Its phosphorylation by Aurora A (AurA) at the basal body of cilia is essential to axonemal depolymerization and ciliary disassembly [22,23,27]. Furthermore, HDAC6 has been implicated in the degradation of non-structural protein (NSP) 8 of PDCoV for inhibiting viral replication [28]. In contrast, both NSP5 proteins of PEDV and PDCoV have been shown to cleave HDAC6 to dampen its antiviral activity [24]. Nonetheless, the precise molecular interactions between structural proteins of PEDV, SARS-CoV-2, IBV, and PDCoV with AurA or HDAC6 and the potential for HDAC6 to serve as pharmacological targets for CoV infections remain uncertain.

In this study, we observed that infection with PEDV and PDCoV triggered ciliary disassembly and led to the upregulation of the AurA-HDAC6 axis. Furthermore, the inhibition of either AurA or HDAC6 activity effectively blocked this disassembly process. We determined that the M protein of all four CoV genera (PEDV, SARS-CoV-2, IBV, and PDCoV) binds to both AurA and HDAC6. Notably, mutations in the conserved amino acid sequences of the M protein resulted in a loss of binding affinity to both AurA and HDAC6, which correspondingly diminished the ability to induce ciliary disassembly. Overall, these findings provide new insights into the interactions between CoVs and host cells, particularly highlighting the critical role of the AurA-HDAC6 axis in mediating ciliary disassembly during CoV early infections. This research not only enhances our understanding of ciliary dynamics in the context of viral pathogenesis but also may inform future therapeutic strategies to mitigate the effects of CoVs on cellular function.

## Results

### Porcine CoVs early infection impairs cilium

**Fig 1:**
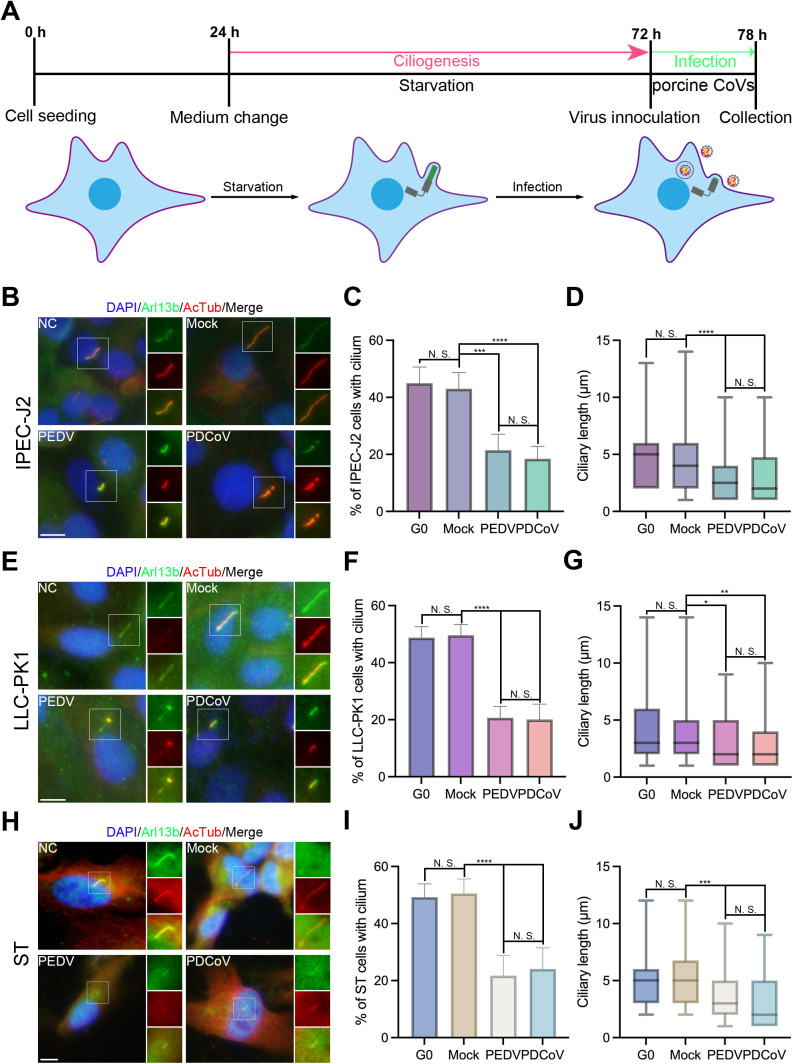
PEDV and PDCoV infection impairs the cilium. (*A*) Flowchart depicting cell seeding, starvation, and subsequent virus inoculation. Model indicating ciliary changes. Immunostaining results for starved intestinal porcine epithelial cell line-J2 (IPEC-J2) (*B*), Lilly Laboratories Cell (LLC)-porcine kidney-1 (PK1) (*E*), and swine testis (ST) (*H*) cells using anti- ADP ribosylation factor-like protein 13b (Arl13b) and acetylated α-tubulin (AcTub) antibodies. (*C-D*) Statistical analysis of results depicted in (*B*). (*F-G*) Statistical analysis of results in (*E*). (*I-J*) Statistical analysis of results in (*H*). Notably, infection by either porcine epidemic diarrhea virus (PEDV) or porcine deltacoronavirus (PDCoV) resulted in a significant decrease in both the percentage of cells with cilium and the average ciliary length across all three porcine cell lines (*C-D, F-G, and I-J*). (*B*, *E*, and *H*), insets (right) displayed magnified views of cilium with separated channels, DNA was stained with DAPI, and Scale bars represented 10 μm. For means (SD) in (*C-D, F-G,* and *I-J*), over 50 cells were counted per replicate from three independent experiments for analysis.

To evaluate whether infection with porcine CoVs similarly induces ciliary disassembly as SARS-CoV-2, intestinal porcine epithelial cell (IPEC)-J2, Lilly Laboratories Cell (LLC)-porcine kidney-1 (PK1), and swine testis (ST) cells were initially starved for 48 hours to promote ciliogenesis [29]. Following starvation, these cells were infected with PEDV and PDCoV at a multiplicity of infection (MOI) of 1 for 6 hours (Fig 1A). To assess ciliary structure, ADP ribosylation factor-like protein 13b (Arl13b) and acetylated α-tubulin (AcTub) antibodies were employed for immunostaining. In comparison to the Mock group, infections with both types of porcine CoVs induced a highly significant reduction in the percentage of cells exhibiting a cilium, decreasing from over 40% to under 20%, as well as a notable decrease in ciliary length from approximately 4–5 μm to about 2 μm on average. Additionally, no significant difference was noted in the percentage of cells with a cilium or ciliary length between the infections caused by PEDV and PDCoV (Fig 1B–1J). Collectively, these data indicate that early infection with porcine CoVs impairs ciliary structure in IPEC-J2 cells, which are derived from host-susceptible organs, as well as in LLC-PK1 and ST cells, which PEDV or PDCoV readily infects (Fig 1A).

### Infection with porcine CoVs promotes AurA-HDAC6 activation

To identify the exact proteins of porcine CoVs that participate in inducing ciliary disassembly, we cloned the structural protein genes of the AH2012/12 strain of PEDV or CZ/2020 strain of PDCoV into the pCDNA3.1 vector with a green fluorescent protein (GFP) tag at the C-terminal. IPEC-J2 cells were transfected with cloned plasmids and GFP-vector. Following transfection, the immunoprecipitated proteins were analyzed and quantified using mass spectrometry (MS) analysis. Several host proteins were found to interact with the PEDV or PDCoV M protein. Among these proteins, HDAC6 binds to PEDV M protein, and AurA associates with PDCoV M protein (Tables 1 and 2).

**Table 1:** A list of the top 10 proteins interacting with the PEDV M protein.

Protein ID	M/vector abundance ratio	Unique Peptide Number	Description
P02543	20.35	53	Vimentin
I3LRS5	9.74	40	Aldehyde dehydrogenase 1 family member A1
A0A5G2QXD8	9.13	36	Nucleolin
Q8WNW3	9.03	24	Junction plakoglobin
A0A287A4W2	8.32	22	Elongation factor for RNA polymerase II
A0A286ZT11	5.97	16	Histone deacetylase 6
A0A287AST1	5.72	13	Opioid growth factor receptor
F2Z5F8	5.72	11	Mediator of ErbB2-driven cell motility
F1RR54	5.48	10	Pre-mRNA-splicing factor
F1RJ01	5.37	9	High mobility group protein B2

**Table 2:** A list of the top 10 proteins interacting with the PDCoV M protein.

Protein ID	M/vector abundance ratio	Unique Peptide Number	Description
P10173	110.73	3	Fumarate hydratase, mitochondrial
Q29361	90.36	9	60S ribosomal protein L35
P68137	58.03	8	Actin, alpha skeletal muscle
Q7SIB7	56.47	13	Phosphoglycerate kinase 1
P62901	55.37	10	60S ribosomal protein L31
Q2EN76	52.71	9	Nucleoside diphosphate kinase B
P62863	41.63	4	40S ribosomal protein S30
P80031	40.54	3	Glutathione S-transferase P
A5GFW1	34.12	6	Aurora kinase A
Q29068	34.00	2	T-complex protein 1 subunit gamma

**Fig 2:**
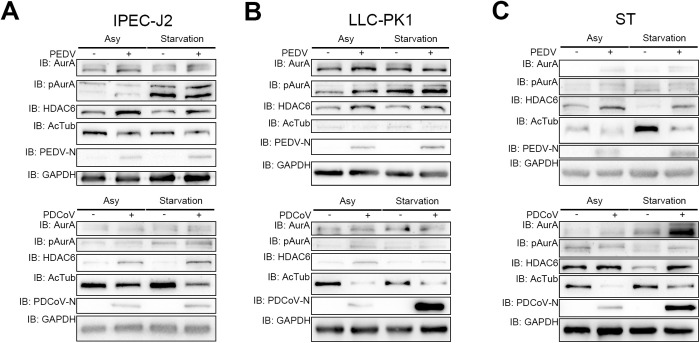
PEDV and PDCoV infection promote the AurA-HDAC6 axis. Immunoblotting results for IPEC-J2 (*A*), LLC-PK1 (*B*), and ST (*C*) cell lysates. Changes in phospho-Aurora A (AurA) -T288 (pAurA) or AcTub protein levels indicate the activation status of AurA or histone deacetylase 6 (HDAC6), respectively. The nucleocapsid (N) protein of PEDV or PDCoV (PEDV-N or PDCoV-N) was utilized as a viral marker, while glyceraldehyde-3-phosphate dehydrogenase (GAPDH) served as a housekeeping control.

Given the established role of the AurA-HDAC6 axis in the depolymerization and disassembly of the ciliary axoneme [23,27], we speculated that ciliary disassembly induced by porcine CoVs is regulated by this axis. To evaluate this hypothesis, we infected both asynchronized (Asy) or starved IPEC-J2, LLC-PK1, and ST cells with PEDV or PDCoV. Our findings revealed that HDAC6, which had been downregulated following starvation, exhibited upregulation after infection with both types of porcine CoV (Fig 2A–2C). Notably, PDCoV infection triggered a greater upregulation in starved IPEC-J2 cells compared to the Asy group (Fig 2A). Moreover, we assessed AcTub protein levels as an indicator of HDAC6 functionality and found that infection with porcine CoVs led to a decrease in AcTub levels, consistent with the observed changes in HDAC6 expression. Additionally, we evaluated the levels of AurA, the upstream kinase of HDAC6. Our results demonstrated that PEDV infection increased AurA expression in Asy cells but not in starved cells, whereas PDCoV infection did not affect AurA expression except in ST cells. Furthermore, using a phospho-AurA-T288 (pAurA) antibody to reflect AurA activation [30], we noted elevated pAurA levels following both porcine CoV infection (Fig 2A–2C).

In general, these results indicate that porcine CoV infection upregulates HDAC6 and activates both AurA and HDAC6, thereby facilitating the activation of the AurA-HDAC6 axis.

### The AurA-HDAC6 axis controls porcine ciliary disassembly

**Fig 3:**
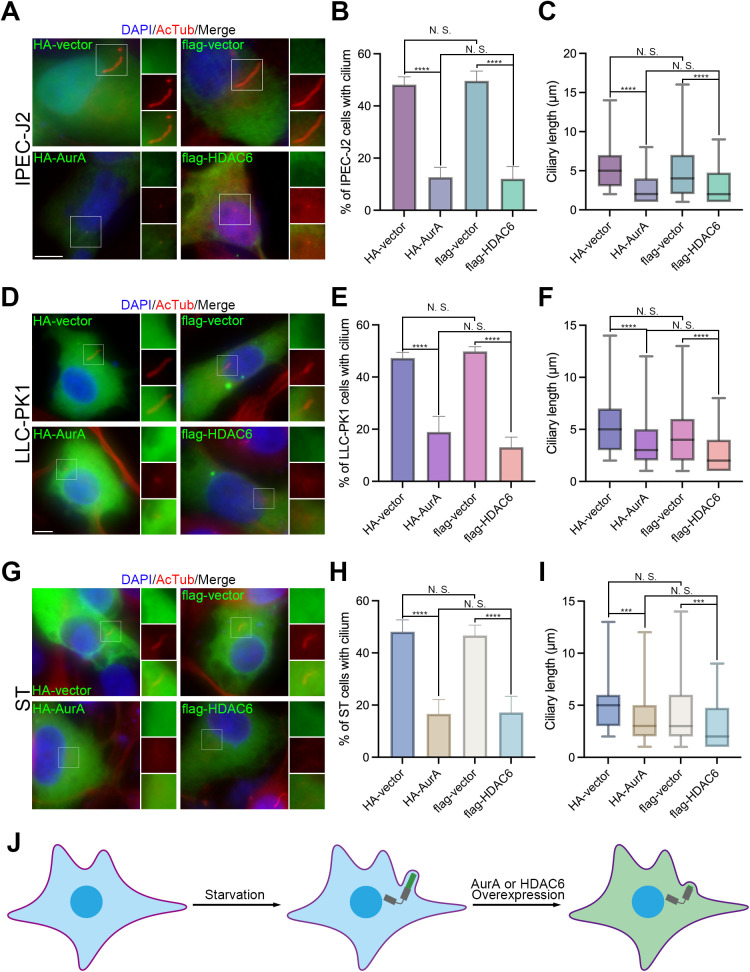
Overexpression of AurA or HDAC6 induces ciliary disassembly. Immunostaining results for starved IPEC-J2 (*A*), LLC-PK1 (*B*), and ST (*C*) cells overexpressing hemagglutinin (HA)- or polypeptide protein (flag)-tagged proteins using AcTub antibodies. (*B-C*) Statistical analysis of results in (*A*). (*E-F*) Statistical analysis of results in (*D*). (*H-I*) Statistical analysis of results in (*G*). (*J*) Model indicating ciliary changes. It is noteworthy that overexpressing either HA-AurA or flag-HDAC6 led to a significant reduction in both the percentage of cells with cilium and the average ciliary length across all porcine cell lines. (*J*) It is noteworthy that overexpressing either HA-AurA or flag-HDAC6 led to a significant reduction in both the percentage of cells with cilium and the average ciliary length across all porcine cell lines. (*A*, *D*, and *G*), insets (right) displayed magnified views of cilium with separated channels, DNA was stained with DAPI, and Scale bars represented 10 μm. For means (SD) in (*C-D* and *F-G*), over 50 cells were respectively counted per replicate from three independent experiments for analysis (*B-C, E-F,* and *H-I*).

To elucidate the function of the AurA-HDAC6 axis on ciliary disassembly in porcine cells, we overexpressed porcine AurA and HDAC6, tagged with hemagglutinin (HA) and polypeptide protein (flag), respectively, in IPEC-J2 (Fig 3A–3C), LLC-PK1 Fig 3D–3F), and ST cells Fig 3G–3I). Utilizing AcTub for ciliary immunostaining, we observed that both overexpressed AurA and HDAC6 resulted in a dramatically significant reduction in the percentage of cells with cilium, decreasing from over 40% to below 20%, and a reduction in ciliary length from approximately 5 μm to 3 μm or less, in comparison to the vector control. Notably, no significant difference was observed between the HA- and flag-tagged vectors or between HA-AurA and flag-HDAC6 (Fig 3). In conclusion, the AurA-HDAC6 axis is implicated in the regulation of ciliary disassembly in porcine cells (Fig 3J).

**Fig 4:**
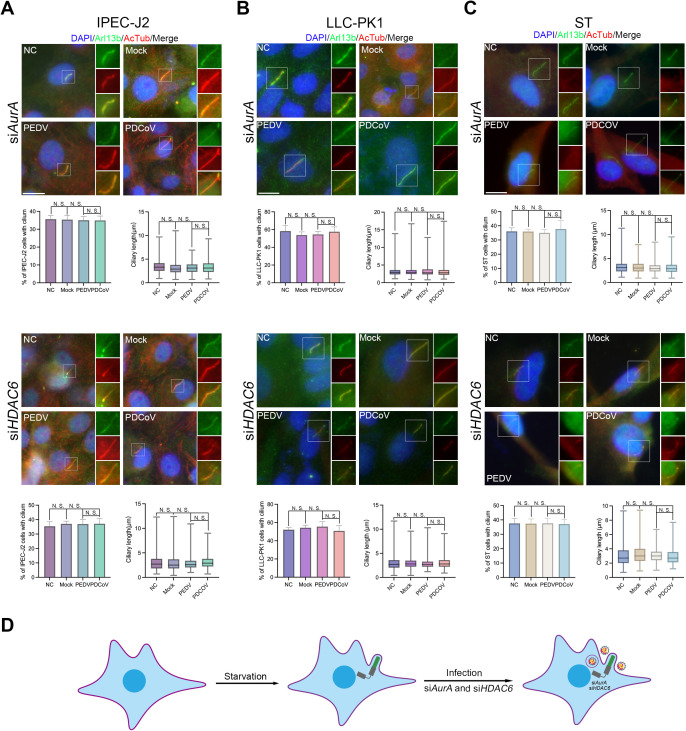
Knockdown of *AurA* or *HDAC6* preserves ciliary structure during porcine CoV infection. Immunostaining results for starved IPEC-J2 (*A*), LLC-PK1 (*B*), and ST (*C*) cells transfected with siHDAC6 or siAurA using Arl13b and AcTub antibodies. (*D*) Model indicating ciliary changes. Both knockdowns effectively reversed the reduction in the percentage of cells exhibiting cilia and ciliary length. (*A-C*) Insets (right) displayed magnified views of cilium with separated channels, DNA was stained with DAPI, and Scale bars represented 10 μm. For means (SD) in (*A-C*), over 50 cells were counted per replicate from three independent experiments for analysis.

It is noteworthy that additional regulatory axes, such as NIMA-like kinase 2 (Nek2)-kinesin family member 24 (Kif24), may also influence ciliary disassembly [31]. To investigate whether the AurA-HDAC6 axis serves as the primary regulator of ciliary disassembly induced by porcine CoV infection, we transfected cells with small interfering RNAs (siRNAs), which were selected for effectiveness (S1 Fig), to achieve knockdown of *AurA* or *HDAC6*. Additionally, cells were treated with specific inhibitors of AurA or HDAC6, namely PHA-680632 (PHA) [32] or Tubacin [33] (S2 Fig). Measurements of pAurA and AcTub revealed that PHA treatment significantly reduced pAurA levels in IPEC-J2 cells, while minor effects were noted in LLC-PK1 and ST cells. Conversely, Tubacin treatment markedly enhanced α-tubulin acetylation across all cell types, demonstrating that both inhibitors effectively restrict AurA and HDAC6 activity (S2A Fig). Subsequent analysis of the percentage of cells exhibiting cilium and ciliary length with knockdown of *AurA*/*HDAC6* (Fig 4A–4C) or under Tubacin/PHA (S2B–S2G Fig) treatment showed that the percentage of ciliated cells remained above 35% and statistically unchanged, regardless of the knockdown or treatment applied. Furthermore, there was no significant difference in ciliary length either. Overall, these data indicate that inhibition of either AurA or HDAC6 activity sufficiently mitigates the impact of porcine CoV infection on ciliary disassembly (Figs 4D and S2H) and that the AurA-HDAC6 axis is identified as the critical regulator of ciliary disassembly in the context of porcine CoV infection.

### Porcine CoV M proteins bind to both AurA and HDAC6 simultaneously

**Fig 5:**
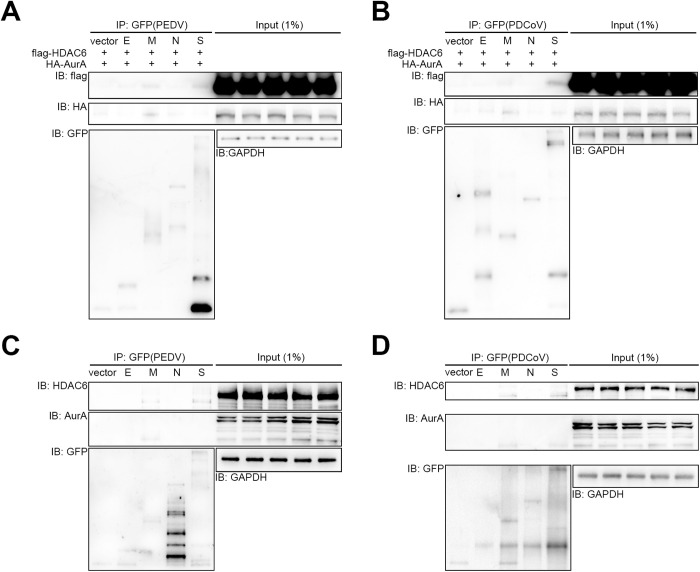
PEDV and PDCoV M proteins interact with AurA and HDAC6 simultaneously. Immunoprecipitation of human embryonic kidney (HEK) 293T cell lysates expressing HA-AurA, flag-HDAC6, and green fluorescent protein (GFP)-tagged PEDV (*A*) or PDCoV (*B*) structural proteins, followed by immunoblotting for HA and flag, demonstrating interactions between the membrane (M) proteins of PEDV or PDCoV with HA-AurA and flag-HDAC6. Immunoprecipitation of IPEC-J2 cell lysates expressing GFP-tagged PEDV (*C*) or PDCoV (*D*) structural proteins, followed by immunoblotting for endogenous AurA and HDAC6, reveals interactions between the M proteins of PEDV or PDCoV with endogenous AurA and HDAC6. GAPDH and GFP levels of overexpressed PEDV or PDCoV structural proteins were utilized as housekeeping controls.

Since we had mapped the binding of AurA or HDAC6 to the PEDV or PDCoV M proteins through MS (Tables 1 and 2), to elucidate further which structural protein in porcine CoV is responsible for binding to AurA and HDAC6, we conducted immunoprecipitation (IP) or co-IP assays in IPEC-J2 and human embryonic kidney (HEK) 293T cells. The results from the co-IP assay indicated that GFP-tagged PEDV M and S proteins bind to flag-tagged porcine HDAC6, with the M protein being the sole entity capable of interacting with HA-tagged porcine AurA (Fig 5A). Similarly, GFP-tagged PDCoV M and S proteins exhibited binding with flag-HDAC6, although the S protein displayed a stronger interaction. Notably, the PDCoV M protein showed a stronger association with HA-AurA than the S protein (Fig 5B). Furthermore, endogenous porcine AurA and HDAC6 demonstrated comparable interaction affinity with GFP-tagged PEDV proteins (Fig 5C). In contrast to the exogenous results, endogenous porcine HDAC6 showed nearly equal binding affinity to both the PDCoV M and S protein, whereas endogenous porcine AurA retained a stronger affinity for PDCoV M protein than for S (Fig 5D). Taken together, these findings indicate that porcine CoV M protein binds with both AurA and HDAC6 simultaneously and may serve as a scaffold.

### The connection region between the TM and cytosolic domains in ciliary disassembly

**Fig 6:**
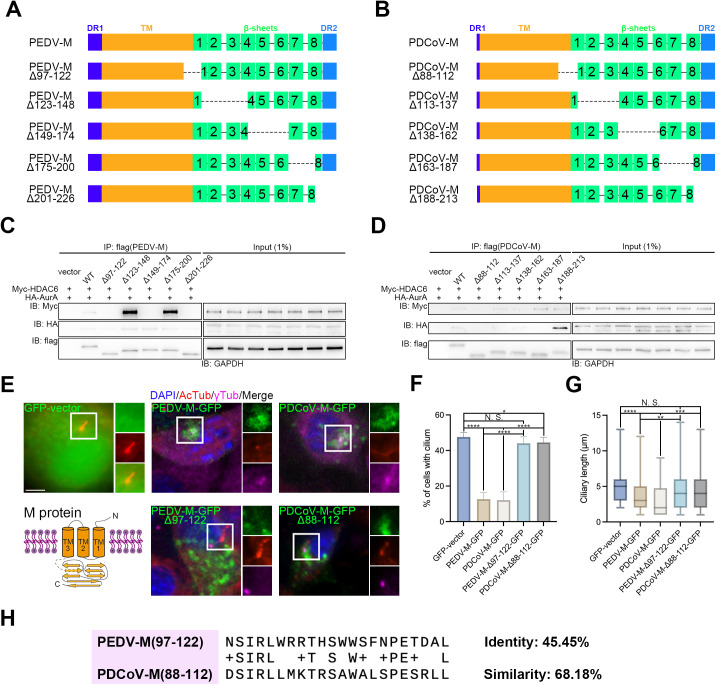
The critical role of the connection region between the TM and the cytosolic domain of porcine CoV M protein in ciliary disassembly. Schematic diagram of full-length or truncated PEDV-M (*A*) and PDCoV-M (*B*) protein structures. Immunoprecipitation of HEK 293T cell lysates expressing Myc-HDAC6, HA-AurA, and flag-tagged wildtype (WT) and PEDV (*C*) or PDCoV (*D*) M protein mutants with deletions within the cytosolic domain, followed by immunoblotting for Myc and HA, illustrating the ablated interactions between the first quintile sequence within the cytosolic domain of PEDV and PDCoV M protein with both Myc and HA. GAPDH and flag levels were used as a housekeeping control (*C-D*). (*E*) Immunostaining of starved IPEC-J2 cells overexpressing WT and PEDV or PDCoV M protein mutants with deletion of the first quintile sequence within the cytosolic domain using AcTub and γ-tubulin (γTub) antibodies. (*F-G*) Statistical analysis of results in (*E*). Note that PEDV or PDCoV M protein mutants with deletions of the first quintile sequence within the cytosolic domain rescued the reduction in both the percentage of cells with cilium and ciliary length induced by the WT (*E-G*). (*H*) Alignment between the protein sequence of PEDV-M(97-122) and PDCoV-M(88-112), with identity and similarity percentages at 45.45% and 68.18%, respectively, conserved Serine (S)99 and Isoleucine (I)100 in PEDV correspond to S89 and I90 in PDCoV. (*E*) Insets (right) displayed magnified views of cilium with separated channels, DNA was stained with DAPI, and scale bars represented 10 μm. For means (SD) in (*F-G*), over 50 cells were counted per replicate from three independent experiments for analysis.

HDAC6 has been implicated in facilitating membrane fusion and uncoating of viral genomic RNA [34]. Following the entry of CoVs, the fusion of endosomal and viral membranes exposes the intravirion domain of the M protein [1]. Consequently, we hypothesized that the crucial region of the M protein responsible for binding to AurA and HDAC6, functioning as a scaffold, lies within the cytosolic domain. To investigate this, we partitioned the cytosolic domain of M proteins from both porcine CoVs, incorporating the last ten amino acids (aa) of their transmembrane (TM) domain to preserve integrity, into five distinct segments (Fig 6A, 6B). Subsequently, we assessed the interactions of these segments with both AurA and HDAC6 through a co-IP assay. For PEDV M protein, deletions (Δ) of aa 123–148 or 175–200 exhibited even higher affinity for porcine HDAC6, whereas Δ97–122, Δ149–174, and Δ201–226 resulted in a loss of this affinity. Notably, only the Δ97–122 failed to bind to porcine AurA (Fig 6C). In the case of PDCoV M protein, Δ88–122 represented the sole mutant unable to interact with porcine HDAC6, while Δ88–112 or Δ163–187 resulted in a loss of binding to porcine AurA. Conversely, Δ188–213 exhibited stronger binding (Fig 6D). In conclusion, aa 97–122 of PEDV M protein and aa 88–112 of PDCoV M protein are defined as the key region for association with the AurA-HDAC6 axis.

Following this, we constructed and transfected GFP-tagged constructs of both the wildtype (WT) and the deletion mutants of the two types of porcine CoV M proteins. Similar to the infection by porcine CoVs (Fig 1), overexpression of the WT PEDV M protein or PDCoV M protein contributed to a marked decrease in the percentage of ciliated cells, declining from approximately 45% in vector-overexpressed cells to below 15%, as well as a reduction in ciliary length from about 5 μm in vector-overexpressed cells to 3 μm or 2 μm, respectively. Conversely, cells overexpressing GFP-tagged PEDV-M(Δ97–122) and PDCoV-M(Δ88–112) proteins exhibited negligible changes in both the percentage of ciliated cells and ciliary length when compared to vector-overexpressed cells. Moreover, we observed that GFP-tagged M proteins of two porcine CoVs aggregated around the centrosome, where cilium protrudes, as indicated by γ-tubulin (γTub). In contrast, PEDV-M(Δ97–122) and PDCoV-M(Δ88–112) failed to exhibit such aggregation (Fig 6E–6G). Furthermore, we aligned protein sequences aa 97–122 of PEDV and aa 88–112 of PDCoV and found that the identity and similarity percentages were 45.45% and 68.18%, respectively (Fig 6H). Consequently, these results indicate that PEDV-M(Δ97–122) and PDCoV-M(Δ88–112) block the effects of porcine CoV M protein on ciliary disassembly.

In summary, we have demonstrated that aa 97–122 of PEDV M protein and aa 88–112 of PDCoV M protein (Fig 6E, dotted part of model) are responsible for regulating ciliary disassembly.

### M proteins of CoVs share conserved interaction sites for binding with the AurA-HDAC6 axis

To determine the exact interaction sites between porcine CoVs M protein and AurA or HDAC6, we performed molecular docking (MD), which computationally predicts the interaction between two candidates [35]. We subsequently screened the most plausible sites through molecular mechanics/generalized born surface area (MM/GBSA) free energy decomposition analysis [36]. For the PEDV M protein (S3A Fig), the top hits for binding to AurA in the region of aa 97–122 were SER(S)108, TRP(W)110, PRO(P)114, and GLU(E)115 (S3B Fig), while the residues binding to HDAC6 included S99 and ILE(I)100 (S3C Fig). In the case of PDCoV M protein (S4A Fig), the top hits for binding to AurA were S98, W100, P104, and E105 (S4B Fig), whereas the residues for HDAC6 were S89 and I90 (S4C Fig). Notably, the two porcine CoV M proteins share identical predicted residues in binding with AurA and HDAC6.

**Fig 7:**
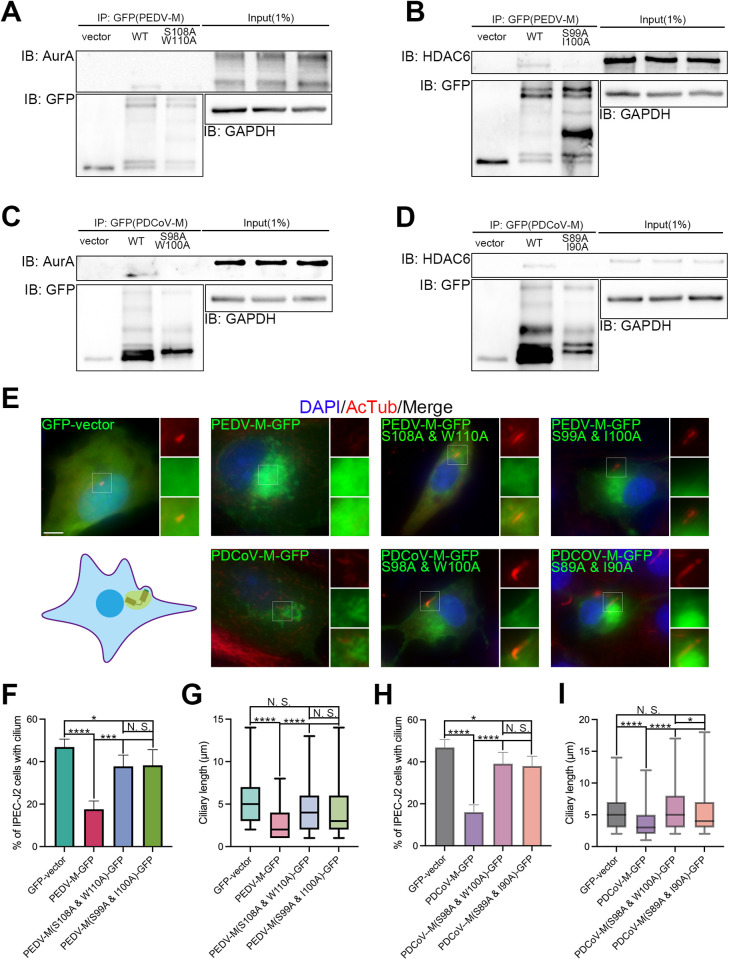
Loss of binding with AurA or HDAC6 blocks porcine CoV M protein-induced ciliary disassembly. Immunoprecipitation of IPEC-J2 cell lysates expressing GFP-tagged PEDV (*A*-*B*) or PDCoV (*C*-*D*) WT and PEDV or PDCoV M protein mutants, followed by immunoblotting for endogenous AurA (*A* and *C*) and HDAC6 (*B* and *D*), illustrating the ablated interactions between the PEDV and PDCoV M mutant proteins with both AurA and HDAC6. GAPDH and GFP levels were utilized as housekeeping controls (*A-D*). (*E*) Immunostaining of starved IPEC-J2 cells overexpressing WT and PEDV or PDCoV M protein mutants using AcTub as a ciliary marker. Model representing an aggregation of M protein around the ciliary basal body (mother centriole) area. Note that PEDV or PDCoV M protein mutants rescued the reduction in both the percentage of cells with cilium and ciliary length induced by the WT. (*F-I*) Statistical analysis of results in (*E*). (*E*) Insets (right) displayed magnified views of cilium with separated channels, DNA was stained with DAPI, and scale bars represented 10 μm. For means (SD) in (*F-I*), over 50 cells were counted per replicate from three independent experiments for analysis.

Considering the binding preference of AurA [37], we constructed S108A/W110A and S99A/I100A mutants of GFP-tagged PEDV M protein (Fig 7A, 7B), along with S98A/W100A and S89A/I90A mutants of GFP-tagged PDCoV M (Fig 7C, 7D) to investigate the specific interaction sites with AurA or HDAC6. IP assays in IPEC-J2 cells demonstrated that PEDV-M(S108A/W110A) and PEDV-M(S99A/I100A) exhibited a significant decrease in binding to AurA and HDAC6, respectively, while PDCoV-M(S98A/W100A) and PDCOV-M(S89A/I90A) completely lost their binding ability (Fig 7A–7D). Following this, we evaluated the impacts of these mutations in comparison with the WT PEDV M protein and PDCoV M protein on ciliary disassembly. Similar to PEDV-M(Δ97–122) and PDCoV-M(Δ88–112) (Fig 7E), overexpression of all four mutants blocked the effects of porcine CoV M protein on ciliary disassembly (Fig 7E–7I), with the percentage of ciliated cells rising back to over 35% from about 15% (Fig 7E-7F and 7H), and ciliary length showing no significant difference (Fig 7G and 7I). However, slight significance was observed between mutants and WT of two porcine CoVs Fig 7F and 7H), and between PDCoV-M(S98A/W100A) and PDCoV-M(S89A/I90A) (Fig 7I). Moreover, we observed that PEDV-M(S99A/I100A) and PDCoV-M(S89A/I90A) aggregated around the ciliary base, similar to the WT, whereas PEDV-M(S108A/W110A) and PDCoV-M(S98A/W100A) failed to exhibit such aggregation (Fig 7E). Thus, conserved residues S108 and W110 of PEDV M protein, as well as S98 and W100 of PDCoV M protein are responsible for interaction with AurA. Meanwhile, residues S99 and I100 of PEDV M protein, along with S89 and I90 of PDCoV M protein, are accountable for binding to HDAC6.

**Fig 8:**
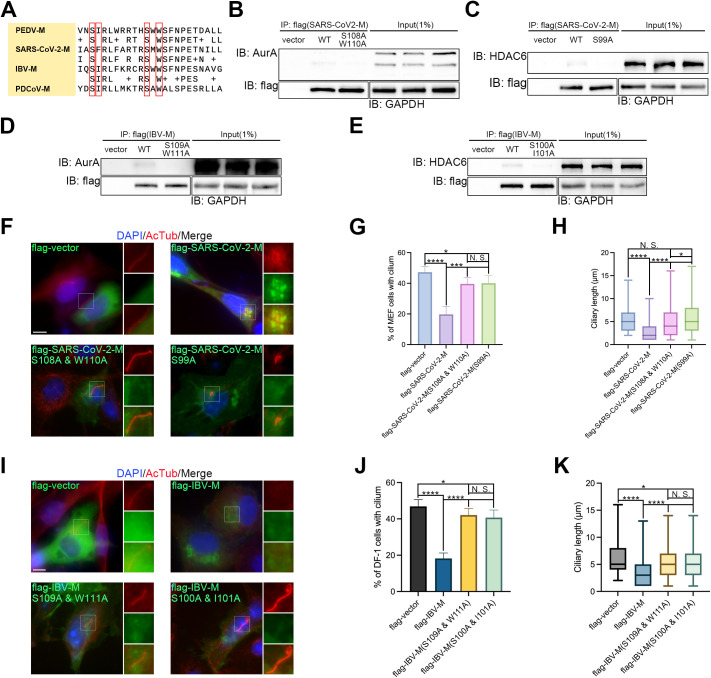
M protein of CoVs shares a common regulatory function on ciliary disassembly through binding with the AurA-HDAC6 axis. (*A*) The aligned protein sequence of the connection regions from the M proteins of PEDV, severe acute respiratory syndrome coronavirus 2 (SARS-CoV-2), infectious bronchitis virus (IBV), and PDCoV. Four genera of CoVs share reserved sites indicated in the red frame. Immunoprecipitation of HEK 293T (*B*-*C*) and Douglas Foster (DF)-1 (*D*-*E*) cell lysates expressing flag-tagged WT and SARS-CoV-2 or IBV M protein mutants, followed by immunoblotting for endogenous AurA (*B* and *D*) and HDAC6 (*C* and *E*), illustrating the ablated interactions between the SARS-CoV-2 and IBV M mutant proteins with both AurA and HDAC6 (*B-E*). GAPDH and flag levels were utilized as housekeeping controls. Immunostaining of starved mouse embryonic fibroblast (MEF) (*F*) or DF-1 (*I*) cells overexpressing WT and SARS-CoV-2 (*F*) or IBV (*I*) M protein mutants using AcTub as a ciliary marker. Note that SARS-CoV-2 or IBV M protein mutants rescued the reduction in both the percentage of cells with cilium and ciliary length induced by the WT (*F* and *I*). (*G*-*H*) Statistical analysis of results in (*G*). (*J*-*K*) Statistical analysis of results in (*I*). (*F* and *I*) Insets (right) displayed magnified views of cilium with separated channels, DNA was stained with DAPI, and scale bars represented 10 μm. For means (SD) in (*G*-*H* and *J*-*K*), over 50 cells were counted per replicate from three independent experiments for analysis.

Since AurA [38] and HDAC6 [39] were also found proximal to the SARS-CoV-2 M protein in proteome research, we subsequently aligned the M proteins of PEDV, SARS-CoV-2, IBV, and PDCoV, which are from all four genera of CoV. We found that both residues S98, S108, and W110 of SARS-CoV-2 M protein, and residues S100, I101, S109, and W111 of IBV M protein are also conserved with PEDV and PDCoV (Fig 8A). Following this, we investigated whether M proteins of SARS-CoV-2 and IBV interact with AurA and HDAC6, and constructed flag-tagged M proteins of SARS-CoV-2 and IBV. IP assays in mouse embryonic fibroblast (MEF) and Douglas Foster (DF)-1 cells demonstrated that M proteins of both SARS-CoV-2 (S5A Fig) and IBV (S5B Fig) bind to AurA and HDAC6. Moreover, similar to PEDV and PDCoV (Figs 5A and S6A), the conserved residues of M proteins from both SARS-CoV-2 (S6A Fig) and IBV (S7A Fig) are located in the connection region between the TM and cytosolic domains as well. To further screen the specific interaction sites between M proteins of SARS-CoV-2 and IBV, we performed MD and MM/GBSA free energy decomposition analysis. For the SARS-CoV-2 M protein, the top hits for binding to AurA were S108 and W110 (S6B Fig), while the residue for HDAC6 was S99 (S6C Fig). In the case of IBV M protein, due to the only member that could be mapped of the chicken Aurora family being Aurora B (AurB) and no HDAC6 could be mapped, we selected AurA and HDAC6 of the Nicobar pigeon, the closest evolutionary relative of chicken we can find as also an MD candidate. The alignment between the protein sequence of chicken AurB and Nicobar pigeon AurA showed the identity and similarity percentages at 82.62% and 90.00%, respectively (S7B Fig). The top hits for binding to chicken AurB and Nicobar pigeon AurA were S109 and W111 (S7C Fig) and S109 (S7D Fig), respectively, while the residues for Nicobar pigeon HDAC6 were S100 and I101 S7E Fig. Thereafter, we constructed S108A/W110A and S99A mutants of flag-tagged SARS-CoV-2 M protein (Fig 8B–8C), along with S109A/W110A and S100A/I101A mutants of flag-tagged IBV M (Fig 8D–8E), to investigate the specific interaction sites with AurA or HDAC6. IP assays in MEF and DF-1 cells demonstrated that SARS-CoV-2-M(S108A/W110A) (Fig 8B), SARS-CoV-2-M(S99A) (Fig 8C), and IBV-M(S109A/W111A) (Fig 8D) nearly completely lost their binding to AurA and HDAC6, while IBV-M(S100A/I101A) (Fig 8E) exhibited a significant decrease in binding ability. Afterward, we evaluated the impacts of these mutations in comparison with the WT SARS-CoV-2 M protein and IBV M protein on ciliary disassembly. Overexpression of all four mutants blocked the effects of WT M proteins on ciliary disassembly (Fig 8F–8K), with the percentage of ciliated cells rising back to around 40% from less than 20% (Fig 8F–8G and 8I, 8J), and ciliary length showing no significant difference (Fig 8H) or slight significance (Fig 8K). However, a slight significance of ciliated cells was observed between mutants and WT for both SARS-CoV-2 (Fig 8G) and IBV (Fig 8J). Furthermore, we observed that SARS-CoV-2-M(S99A) and IBV-M(S100A/I101A) aggregated around the ciliary base, similar to the WT M proteins of SARS-CoV-2, IBV, PEDV, and PDCoV (Figs 6E, 7E, 8F, and 8I), whereas SARS-CoV-2-M(S108A/W110A) and IBV-M(S109A/W111A) failed to exhibit such aggregation (Fig 8F and 8I). Hence, conserved residues S108 and W110 of SARS-CoV-2 M protein, as well as S109 and W111 of IBV M protein, are responsible for interaction with AurA. Meanwhile, residue S99 of SARS-CoV-2 M protein, along with S100 and I101 of IBV M protein, is accountable for binding to HDAC6.

**Fig 9:**
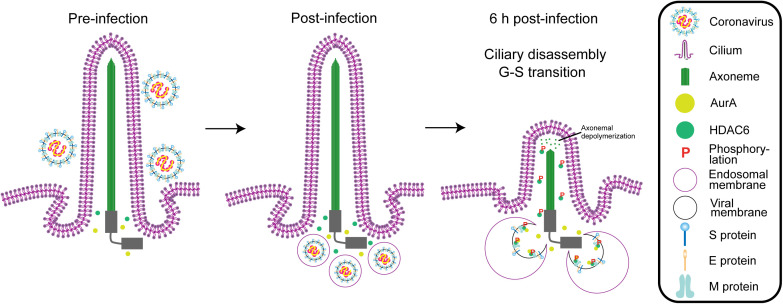
Schematic representation of the mechanism by which porcine CoVs infection impairs ciliary structure. A working model outlines the roles of the M protein of CoVs. Before infection, AurA and HDAC6 accumulate around the ciliary base area. During the initial stages of infection, upon entry into host cells and fusion of the endosomal and viral membranes, the cytosolic domain of the M proteins is rapidly exposed to the cytoplasm, facilitating interactions with AurA and HDAC6 as a scaffold, which in turn triggers phosphorylation of HDAC6 by AurA and ciliary translocation of phosphorylated HDAC6. Ciliary HDAC6 subsequently regulates axonemal depolymerization and ciliary disassembly.

Above all, CoV M proteins of all four genera share a common regulatory function on ciliary disassembly through binding with the AurA-HDAC6 axis (Fig 9).

## Discussion

CoVs represent a large family of viruses known to infect a diverse variety of mammalian and avian species [1,40]. However, the mechanisms underlying the early infection by CoVs on the cilium remain unclear. This study employed MS, FC, IP, MD, and MM/GBSA analyses to identify the M protein of all four CoV genera as a crucial mediator in regulating ciliary disassembly through the AurA-HDAC6 axis. We demonstrated that CoV M proteins impair cilium by binding to AurA and HDAC6 as a scaffold, pinpointing specific conserved residues in the connection region between the TM and cytosolic domains responsible for these interactions. Mutations in these residues significantly obstructed binding to AurA and HDAC6, consequently blocking ciliary disassembly (Fig 9). This model underscores a common regulatory function of CoV M proteins across genera, thereby partially elucidating the potential mechanisms underlying the damage inflicted by CoVs on specific organs. Significant findings include the detrimental effects observed in intestinal epithelial cells of piglets resulting from PEDV [41,42] and PDCoV [8,43] infections, as well as the cessation of ciliary movement in the tracheal epithelial cells of chicks following IBV infection [20]. Furthermore, this study provides a new perspective for exploring the interaction mechanisms between CoVs and hosts, paving the way for future research in this critical area, and informs the development of a framework to identify key modulators of the host antiviral response.

HDACs are implicated in viral infections; for instance, Marek’s disease virus (MDV) interacts with HDAC1 and HDAC2 to facilitate their degradation [44], while the knockout of *HDAC9* enhances foot-and-mouth disease virus (FMDV) replication [45]. HDAC6 has been shown to inhibit the release of influenza A virus (IAV) [46], degrade the NSP5 of Zika virus (ZIKV) [25], and trigger the DNA damage response to promote the replication of pseudorabies virus (PRV) [47]. The role of HDAC6 in ciliary disassembly has been extensively studied over the past decades [22,27,48,49], and increasing attention has been directed toward its involvement in viral infections [24,25], including ZIKV [25], IAV [46], porcine reproductive and respiratory syndrome virus (PRRSV) [50], porcine circoviruses 2 (PCV2) [51], and FMDV [52]. Thus, HDAC6 serves not only as a viral target but also as a regulator of the viral infection process. Our findings are consistent with recent studies indicating that elevated HDAC6 expression levels resulted in reduced levels of AcTub. The observed discrepancy between the upregulation of HDAC6 at 6 hours post-infection with PEDV or PDCoV in our study (Fig 2A–2C) and the downregulation after 12 hours or more post-infection with PDCoV [28] could be attributed to the stage of infection, as our study collected cells at the 6-hour time point, and to the cell type, as the elevated level of HDAC6 was weaker than that observed in IPEC-J2 and ST cells. Furthermore, PEDV, TGEV, PDCoV, and SADS-CoV exhibited nearly equal major bands over 100 kilodaltons (kDa) 12 h post-infection in LLC-PK1 [24]. Given that PDCoV replicated more rapidly in starved LLC-PK1 and ST cells compared to IPEC-J2 cells, the level of HDAC6 elevation was weakened as well (Fig 2A–2C), possibly due to the cleavage function of PDCoV NSP5, aligning with observations from earlier studies. Therefore, our results contribute novel insights into the implications of CoV infections and the role of HDAC6 in viral infection, thereby informing future therapeutic strategies aimed at mitigating the effects of CoVs on cellular function.

Infection with both porcine CoVs resulted in a markedly significant reduction in the percentage of ciliated cells and ciliary length in IPEC-J2 cells (Fig 1B–1D) and ST cells (Fig 1H–1J). Conversely, PEDV infection displayed only a marginal significance when compared to the Mock group in LLC-PK1 cells (Fig 1E–1G). This observation may be ascribed to the comparatively weaker infectivity of PEDV in LLC-PK1 cells relative to that in IPEC-J2 or ST cells. Furthermore, levels of AurA were found to be upregulated in all three asynchronized cell types while remaining nearly unchanged in starved cells, except for PDCoV infection in ST cells. Notably, the levels of pAurA in starved cells were significantly elevated compared to the control (Fig 2A–2C), potentially due to quiescent cells activating protective mechanisms to ensure their survival in the absence of growth with low protein expression levels [53,54]. When AurA was inhibited using PHA, the ciliary length was observed to be approximately 1 μm longer than with Tubacin treatment (S2 Fig). This could be because the inhibition of AurA not only influences ciliary disassembly [55] but also disrupts its regulatory role in mitotic progression during the cell cycle [56]. Due to crosstalk between cilia or AurA kinase and cell cycle progression, future investigations should aim to elucidate the relationships among CoV infections, the AurA-HDAC6 axis, and cell cycle progression, thereby enhancing our understanding of the mechanisms governing the switch between ciliary facilitation of viral entry and blockade of cell cycle progression, which is essential for the development of precise drug targets to treat virus-induced diseases.

The M protein of porcine CoVs is classified as one of the structural proteins. Upon entry into host cells and subsequent fusion of the endosomal and viral membranes, the cytosolic domain of the M proteins is rapidly exposed to the cytoplasm, facilitating interactions with AurA and HDAC6, which subsequently trigger ciliary disassembly. In contrast, NSPs remain untranslated at this time point [40,57]. Thus, our study provides a new insight into host-virus interaction during early infection, particularly during endosomal and viral membrane fusion. Furthermore, the M proteins of all four CoV genera are concentrated in the ciliary base area (Figs 6E, 7E, 8F, and 8I), where the basal body, also referred to as the mother centriole, functions as the microtubule-organizing center (MTOC). It has been reported that PEDV is transported along the microtubules [58]. Moreover, HDAC6 has been shown to associate with the microtubule motor protein dynein, facilitating the transport of cargo that aggregates around the MTOC along the microtubule [59]. With the identification of the interaction between the M protein of CoVs and HDAC6, it is plausible that CoVs not only regulate ciliary disassembly but also leverage HDAC6 to facilitate rapid transport from the MTOC to target organelles along the microtubule following infection and membrane fusion. This dual role underscores the potential mechanisms by which CoVs may manipulate host cellular processes to their advantage during the early stages of infection.

Last but not least, this study is subject to several limitations. Firstly, while PDCoV is the only *deltacoronavirus* reported infecting porcine, the addition of TGEV and SADS-CoV, both of which are also porcine CoVs and belong to *alphacoronavirus* genus, would enhance the representativeness of our findings. Secondly, the aa sequence 97–122 of the PEDV M protein contains three serine and four threonine residues, while the PDCoV M protein sequence 88–112 contains four serine and one threonine residue, all of which could potentially be phosphorylated by AurA. Although this connection may not be immediately apparent, future investigations could further explore this phosphorylation mechanism. Finally, despite the significantly decreased binding band of PEDV-M(S108A/W110A) (Fig 7A), PEDV-M(S99A/I100A) (Fig 7B), and IBV-M(S100A/I101A) (Fig 8E) compared to WT, the presence of additional binding sites to AurA or HDAC6 remains unknown.

## Materials and methods

### Cell culture, viruses, and transfection

HEK 293T, MEF, DF-1, IPEC-J2, LLC-PK1, and ST cells came from the American Type Culture Collection (ATCC). Cells were cultured at 37 °C in Dulbecco’s modified Eagle medium (Gibco) and starved with medium containing 0.5% fetal bovine serum (Gibco), 100 U/ml penicillin (Invitrogen), and 100 μg/ml streptomycin (Invitrogen) in the presence of 5% CO_2_.

The AH2012/12 (GenBank accession number: KU646831) strain and PDCoV CZ/2020 strain (GenBank accession number: OK546242) were restored in our laboratories.

According to the manufacturer’s protocol, cells were transfected using HighGene plus Transfection reagent (RM09014P, Abclonal). PHA (HY-10178, MCE) and Tubacin (HY-13428, MCE) were used to inhibit AurA and HDAC6.

### Virus propagation

PEDV and PDCoV were propagated in ST and LLC-PK1 cells, respectively. IPEC-J2, LLC-PK1, and ST cells were cultured in 6-well plates and infected with PEDV or PDCoV, which were diluted in serum-free DMEM at a multiplicity of infection (MOI) of 1 with 10 μg/mL trypsin. After 1 h of absorption at 37 °C, the medium was removed. The infected cells were then washed with PBS three times and cultured in fresh DMEM supplemented with 2% FBS at 37 °C for 5 h.

### Plasmids and siRNA

Porcine AurA (GenBank accession number: XM_005673034.2) and HDAC6 (GenBank accession number: XM_005673600.3) were synthesized and cloned into the pRK5 vector with HA, Myc, or flag tag, respectively. PEDV and PDCoV E, M, N, and S genes were synthesized and cloned into the pCDNA3.1 or pRK5 vector with a GFP tag. PEDV-M(S108A/W110A and S99A/I100A) and PDCoV-M (S98A/W100A and S89A/I90A) were mutated from WT. PEDV-M(WT, Δ97–122, Δ123–148, Δ149–174, Δ175–200, and Δ201–225), SARS-CoV-2-M(WT, S108A/W110A, and S99A/I100A), IBV-M(WT, S109A/W111A, and S100A/I101A), and PDCOV-M(WT, Δ88–112, Δ113–139, Δ138–162, Δ163–187, and Δ188–213) were mutated and cloned into the pRK5 vector with a flag tag. Primers (S1 Table) and siRNAs (S2 Table) used are listed in Supplementary Information.

### IP and Western blotting

Cells were washed with cold PBS and lysed in cell lysis buffer (20 mM Tris-HCl, pH 8.0; 150 mM NaCl; 2 mM EGTA; 0.5 mM EDTA; 0.5% NP-40; 5 mM NaF; 1 mM Na_3_VO_4_; 1 mM PMSF; and 500 × protease inhibitor cocktail; Abclonal) for 20 min on ice. For the flag IP assay, anti-flag M2 magnetic beads (Sigma-Aldrich) were incubated with cell lysates at 4°C for 1 h. For the GFP IP assay, anti-GFP magnetic beads (Abclonal) were incubated with cell lysates at 4°C for 1 h. After washing with lysis buffer, the lysates were harvested in sample buffer and boiled at 95°C for 10 min. After resolution on SDS-PAGE gels, the proteins were transferred to nitrocellulose membranes that were then blocked in TBST (20 mM Tris-HCl, pH 7.5; 500 mM NaCl; and 0.3% Tween 20) containing 1% nonfat milk at room temperature for 1 h. They were probed with indicated primary antibodies and then with horseradish peroxidase (HRP)-conjugated secondary antibody. The membranes were developed using enhanced chemiluminescence (Abclonal) and then exposed to a film machine (Tanon).

### Immunofluorescence

Cells grown on slides were fixed in 4% paraformaldehyde at room temperature for 20 min, permeabilized with 0.1% Triton X-100 for 5 min, and incubated with primary antibodies at 4°C overnight. After a wash in PBS 3 times, the cells were incubated with secondary antibodies for 1 h at room temperature and mounted with Mowiol, mixing 1 mg/mL DAPI for DNA staining. Data were acquired with a spinning disc confocal microscope equipped with an inverted microscope (Nikon TiE) and a 60 × /1.4 or 100 × /1.4 NA oil objective. Images were captured with an EM-CCD camera, and different Z sections were overlaid by Volocity.

### Antibodies

Antibodies were obtained as follows: anti-PEDV-N (M100048) was from Zoonogen (China), anti-PDCoV-N (PDCOV11-M) was from Alpha Diagnostic International (USA), anti- GFP (AE078), DDDDK (AE092), and pAurA (AP0523) were from the Abclonal (China); anti- Arl13b (17711–1-AP) and GAPDH (60004–1-Ig) were from Proteintech (China); anti- AurA (14475), HDAC6 (7558), and HA (3724) were from Cell Signaling Technology (USA); and anti- acetylated α-tubulin (T7451) and γ-Tub (T3559) were from Sigma-Aldrich (China). The secondary antibodies were all obtained from Abclonal (China).

### Protein preparation

Three-dimensional structure models for the M proteins of porcine epidemic diarrhea virus (PEDV), porcine deltacoronavirus (PDCoV), and infectious bronchitis virus (IBV), along with chicken Aurora B (AurB), nicobar pigeon Aurora A (AurA), and nicobar pigeon Histone deacetylase 6 (HDAC6) were built using the SWISS-MODEL server (https://swissmodel.expasy.org/) [60]. Additionally, three-dimensional structure models of human AurA (AF-O14965-F1), human HDAC6 (AF-Q9UBN7-F1), porcine AurA (AF-A5GFW1-F1), and porcine HDAC6 (AF-A0A287BPX4-F1) were predicted via AlphaFold server (https://alphafold.com/) [61,62]. The three-dimensional structure of severe acute respiratory syndrome coronavirus 2 (SARS-CoV-2) M protein (PBDID: 7vgr) was retrieved from Protein Data Bank Europe (PDBe) (https://www.ebi.ac.uk/pdbe/).

### Protein–protein docking

We employed the HDOCK web server (http://hdock.phys.hust.edu.cn/) to conduct MD between the M proteins of PEDV, SARS-CoV-2, IBV, or PDCoV and AurA or HDAC6 [35]. The MM/GBSA free energy decomposition analysis of the HawkDock server (http://cadd.zju.edu.cn/hawkdock/) was utilized subsequently to identify the optimal docking models generated by HDOCK [36]. Finally, the 3D protein–protein interactions were visualized using PyMOL 2.4.

### Quantification and statistical analyses

Statistical testing was performed using GraphPad Prism software.

For the percentage of ciliated cells, *P*-values were calculated from the mean values of the indicated data using Logistic Regression. Data are presented as means (SD).

For ciliary length, *P*-values were calculated from the mean values of the indicated data by One-Way ANOVA. The normality of the data and homogeneity of variances were assessed visually through Q-Q plots of the raw residuals and a plot of studentised residuals versus fitted values, respectively. When these assumptions were violated, the data were log-transformed before analysis to pass the normality test, and if log-transformed, the geometric mean instead of the arithmetic mean was presented (SD).

*P* > 0.05, “N. S.”, *P* < 0.05, “*”, *P* < 0.01, “**”, *P* < 0.001, “***”, *P* < 0.0001, “****”.

## Supporting information

S1 Fig Knockdown of AurA and HDAC6. Immunoblotting of intestinal porcine epithelial cell line-J2 (IPEC-J2) cell lysates transfected with siRNA for Aurora A (AurA) (siAurA) (*A*) or histone deacetylase 6 (HDAC6) (*B*), and irrelevant siNC, with glyceraldehyde-3-phosphate dehydrogenase (GAPDH) as housekeeping control. Note that AurA or HDAC6 was successfully knocked down by siAurA-3 or siHDAC6–2, respectively.
(TIF)

S2 Fig Inhibition of AurA or HDAC6 by inhibitors preserves ciliary structure during porcine CoVs infection. (*A*) Immunoblotting results for IPEC-J2, Lilly Laboratories Cell (LLC)-porcine kidney-1 (PK1), and swine testis (ST) cell lysates using anti- phospho-AurA-T288 (pAurA) and acetylated α-tubulin (AcTub) antibodies, with GAPDH as a housekeeping control. Tubacin treatment increased AcTub levels, while PHA-680632 (PHA) treatment decreased pAurA levels, indicating successful inhibition of AurA and HDAC6. Immunostaining results for Tubacin or PHA-treated starved IPEC-J2 (*B-C*), LLC-PK1 (*D-E*), and ST (*F-G*) cells using ADP ribosylation factor-like protein 13b (Arl13b) and AcTub antibodies. (*H*) Model indicating ciliary changes. Both inhibitor treatments effectively reversed the reduction in the percentage of cells exhibiting cilia and ciliary length. (*B-G*) Insets (right) displayed magnified views of cilium with separated channels, DNA was stained with DAPI, and Scale bars represented 10 μm. For means (SD) in (*B-G*), over 50 cells were counted per replicate from three independent experiments for analysis.
(TIF)

S3 Fig Interaction modeling of PEDV M protein with AurA and HDAC6. (*A*) The protein sequence and structural domains of the porcine epidemic diarrhea virus (PEDV) membrane (M) protein. (*B-C*) Illustrative models of mimic binding conformations between PEDV M protein (violet) and porcine AurA (aquamarine) (*B*) or HDAC6 (marine) (*C*). The mimic interaction sites are shown in an enlarged view and emphasized as model color in (*A*).
(TIF)

S4 Fig Interaction modeling of PDCoV M protein with AurA and HDAC6. (*A*) The protein sequence and structural domains of porcine deltacoronavirus (PDCoV) M protein. (*B-C*) Illustrative models of mimic binding conformations between PDCoV M protein (red) and porcine AurA (aquamarine) (*B*) or HDAC6 (marine) (*C*). The mimic interaction sites are shown in an enlarged view and emphasized as model color in (*A*).
(TIF)

S5 Fig SARS-CoV-2 and IBV M proteins interact with AurA and HDAC6 simultaneously. (*A*) Immunoprecipitation of human embryonic kidney (HEK) 293T cell lysates expressing flag-tagged severe acute respiratory syndrome coronavirus 2 (SARS-CoV-2) M protein, followed by immunoblotting for endogenous AurA and HDAC6, revealing interactions between the SARS-CoV-2 M protein with endogenous human AurA and HDAC6. (*B*) Immunoprecipitation of Douglas Foster (DF)-1 cell lysates expressing flag-tagged infectious bronchitis virus (IBV) M protein, followed by immunoblotting for endogenous chicken AurA and HDAC6, revealing interactions between the IBV M protein with endogenous AurA and HDAC6.
(TIF)

S6 Fig Interaction modeling of SARS-CoV-2 M protein with AurA and HDAC6. (*A*) The protein sequence and structural domains of SARS-CoV-2 M protein. (*B-C*) Illustrative models of mimic binding conformations between SARS-CoV-2 M protein (orange) and human AurA (purple-blue) (*B*) or HDAC6 (forest) (*C*). The mimic interaction sites are shown in an enlarged view and emphasized as model color in (*B-C*).
(TIF)

S7 Fig Interaction modeling of IBV M protein with AurA and HDAC6. (*A*) The protein sequence and structural domains of IBV M protein. (*B*) Alignment between the protein sequence of chicken Aurora B (AurB) and Nicobar pigeon AurA, with identity and similarity percentages at 82.62% and 90.00%, respectively. (*C-E*) Illustrative models of mimic binding conformations between IBV M protein (light orange) and chicken AurB (light blue) (*C*), Nicobar pigeon AurA (pale cyan) (*D*) or HDAC6 (limon) (*E*).
(TIF)

S1 Table Primers used for constructing plasmids. (XLSX)

S2 Table siRNAs used for the knockdown of AurA or HDAC6. (XLSX)

S3 Table Raw data related to Fig 1. (XLSX)

S4 Table Raw data related to Fig 3. (XLSX)

S5 Table Raw data related to Fig 4. (XLSX)

S6 Table Raw data related to Fig 6. (XLSX)

S7 Table Raw data related to Fig 7. (XLSX)

S8 Table Raw data related to Fig 8. (XLSX)

S9 Table Raw data related to S2 Fig. (XLSX)
