# Supplementary figures and images for: Coronavirus M protein impairs cilium during early infection by enhancing the AurA-HDAC6 axis
Source: PLoS Pathog. 2025 Sep 12;21(9):e1013515. doi: 10.1371/journal.ppat.1013515 (PMC12445521; doi:10.1371/journal.ppat.1013515)

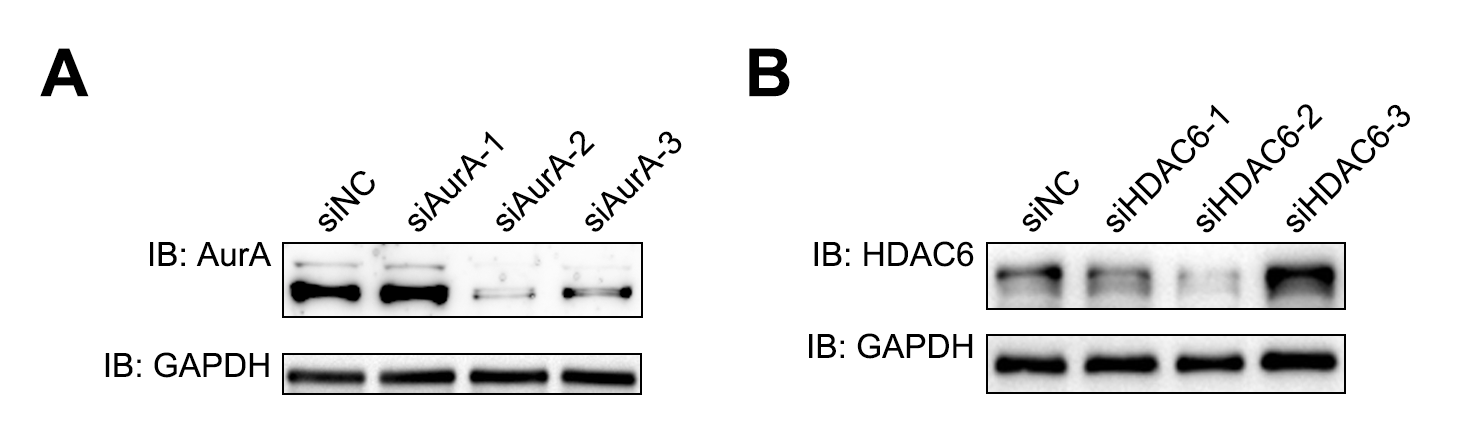

Supplement: S1 Fig — Immunoblotting of intestinal porcine epithelial cell line-J2 (IPEC-J2) cell lysates transfected with siRNA for Aurora A (AurA) (siAurA) (A) or histone deacetylase 6 (HDAC6) (B), and irrelevant siNC, with glyceraldehyde-3-phosphate dehydrogenase (GAPDH) as housekeeping control. Note that AurA or HDAC6 was successfully knocked down by siAurA-3 or siHDAC6–2, respectively. (TIF) [file ppat.1013515.s001.tif]

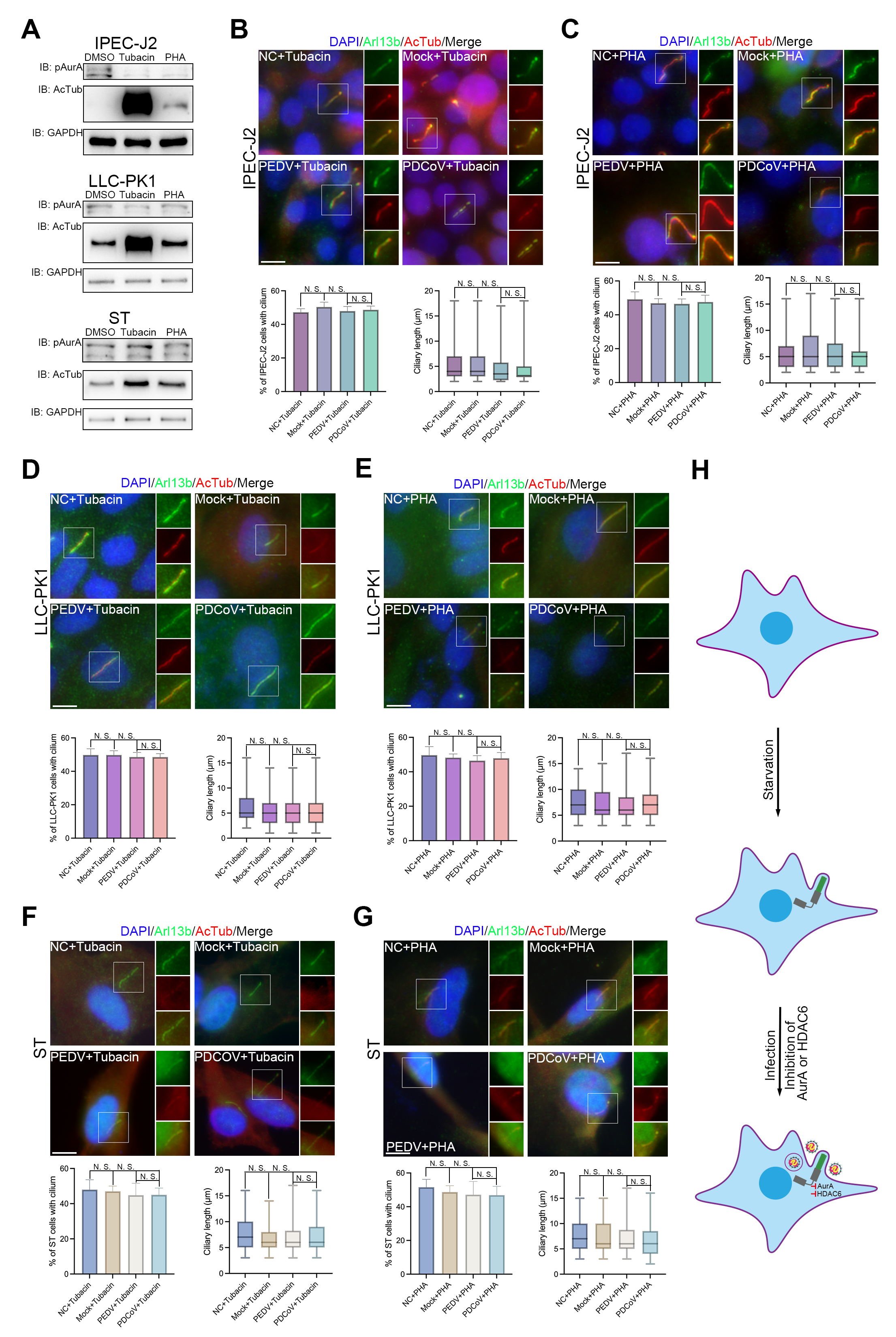

Supplement: S2 Fig — (A) Immunoblotting results for IPEC-J2, Lilly Laboratories Cell (LLC)-porcine kidney-1 (PK1), and swine testis (ST) cell lysates using anti- phospho-AurA-T288 (pAurA) and acetylated α-tubulin (AcTub) antibodies, with GAPDH as a housekeeping control. Tubacin treatment increased AcTub levels, while PHA-680632 (PHA) treatment decreased pAurA levels, indicating successful inhibition of AurA and HDAC6. Immunostaining results for Tubacin or PHA-treated starved IPEC-J2 (B-C), LLC-PK1 (D-E), and ST (F-G) cells using ADP ribosylation factor-like protein 13b (Arl13b) and AcTub antibodies. (H) Model indicating ciliary changes. Both inhibitor treatments effectively reversed the reduction in the percentage of cells exhibiting cilia and ciliary length. (B-G) Insets (right) displayed magnified views of cilium with separated channels, DNA was stained with DAPI, and Scale bars represented 10 μm. For means (SD) in (B-G), over 50 cells were counted per replicate from three independent experiments for analysis. (TIF) [file ppat.1013515.s002.tif]

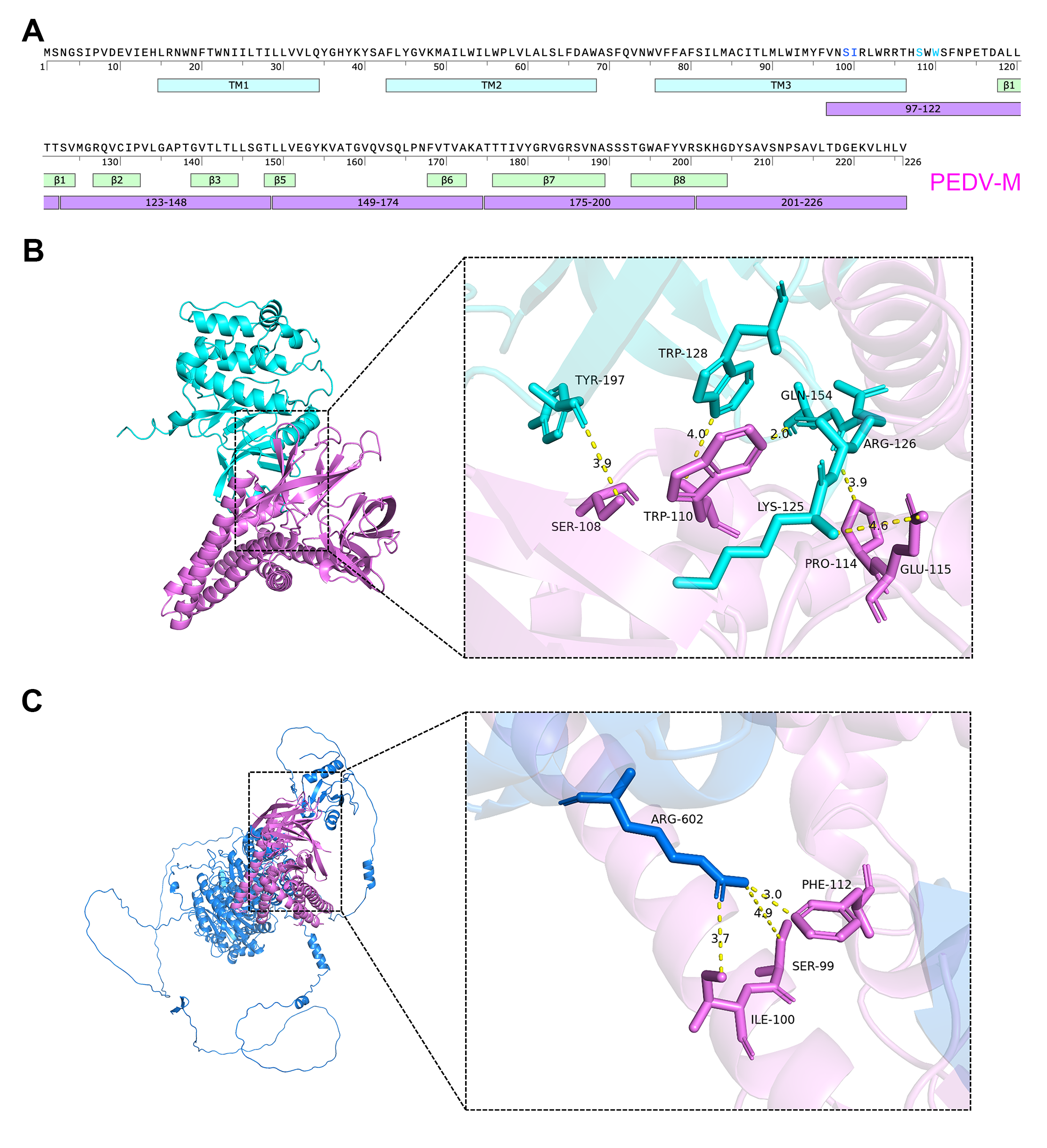

Supplement: S3 Fig — (A) The protein sequence and structural domains of the porcine epidemic diarrhea virus (PEDV) membrane (M) protein. (B-C) Illustrative models of mimic binding conformations between PEDV M protein (violet) and porcine AurA (aquamarine) (B) or HDAC6 (marine) (C). The mimic interaction sites are shown in an enlarged view and emphasized as model color in (A). (TIF) [file ppat.1013515.s003.tif]

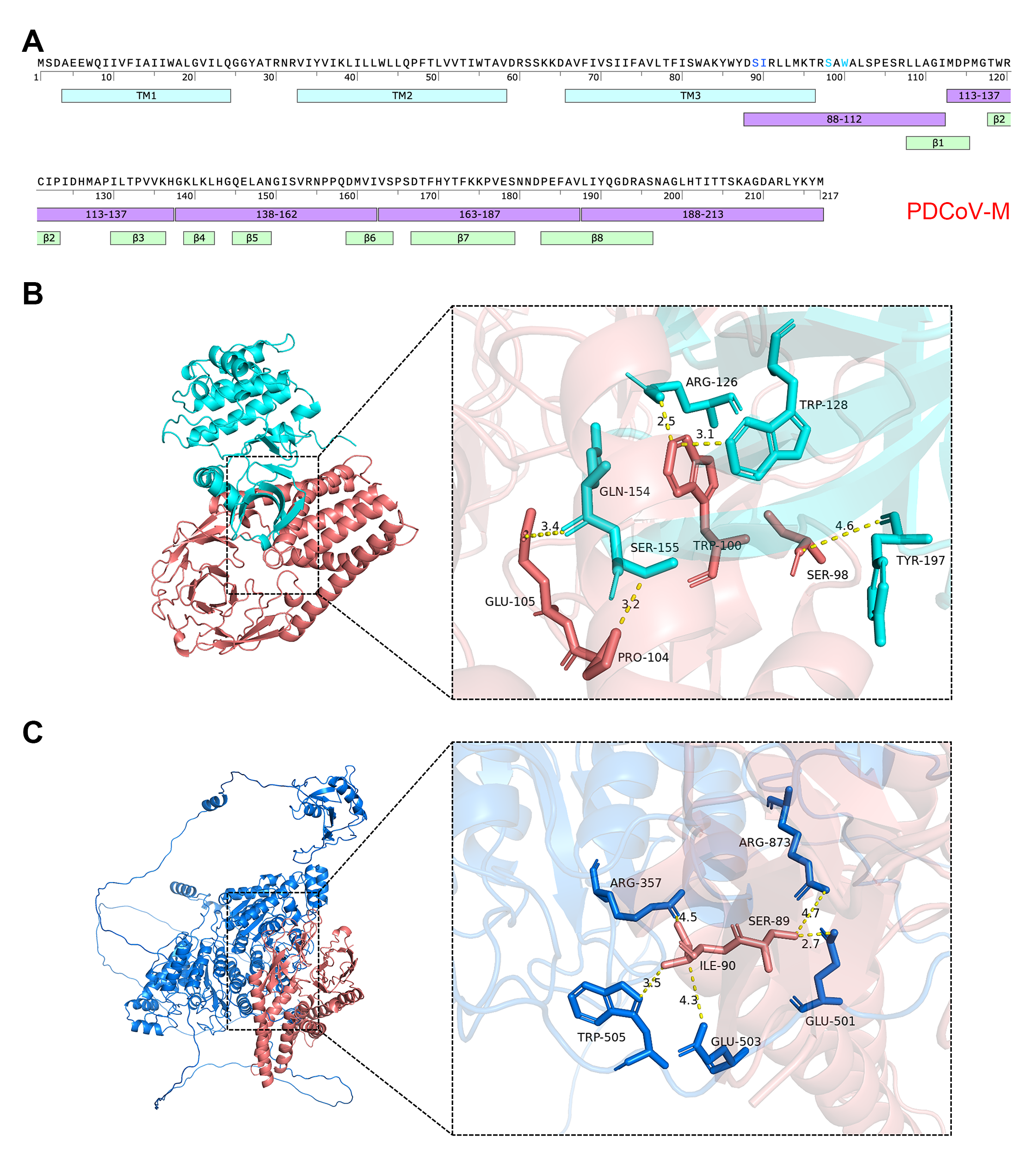

Supplement: S4 Fig — (A) The protein sequence and structural domains of porcine deltacoronavirus (PDCoV) M protein. (B-C) Illustrative models of mimic binding conformations between PDCoV M protein (red) and porcine AurA (aquamarine) (B) or HDAC6 (marine) (C). The mimic interaction sites are shown in an enlarged view and emphasized as model color in (A). (TIF) [file ppat.1013515.s004.tif]

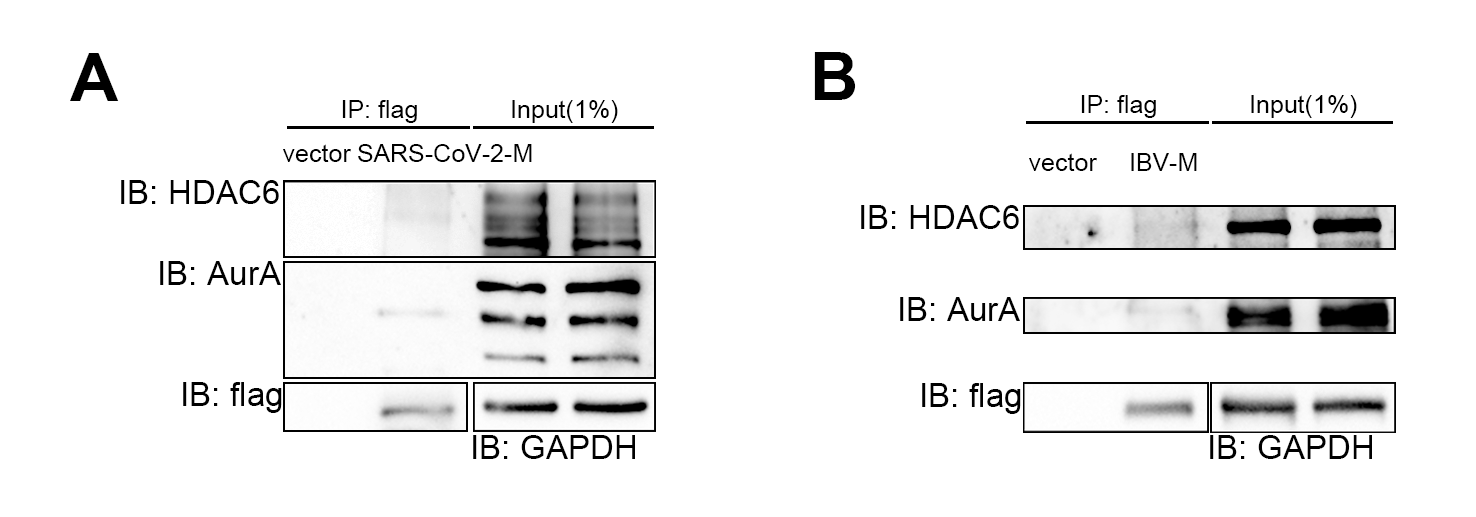

Supplement: S5 Fig — (A) Immunoprecipitation of human embryonic kidney (HEK) 293T cell lysates expressing flag-tagged severe acute respiratory syndrome coronavirus 2 (SARS-CoV-2) M protein, followed by immunoblotting for endogenous AurA and HDAC6, revealing interactions between the SARS-CoV-2 M protein with endogenous human AurA and HDAC6. (B) Immunoprecipitation of Douglas Foster (DF)-1 cell lysates expressing flag-tagged infectious bronchitis virus (IBV) M protein, followed by immunoblotting for endogenous chicken AurA and HDAC6, revealing interactions between the IBV M protein with endogenous AurA and HDAC6. (TIF) [file ppat.1013515.s005.tif]

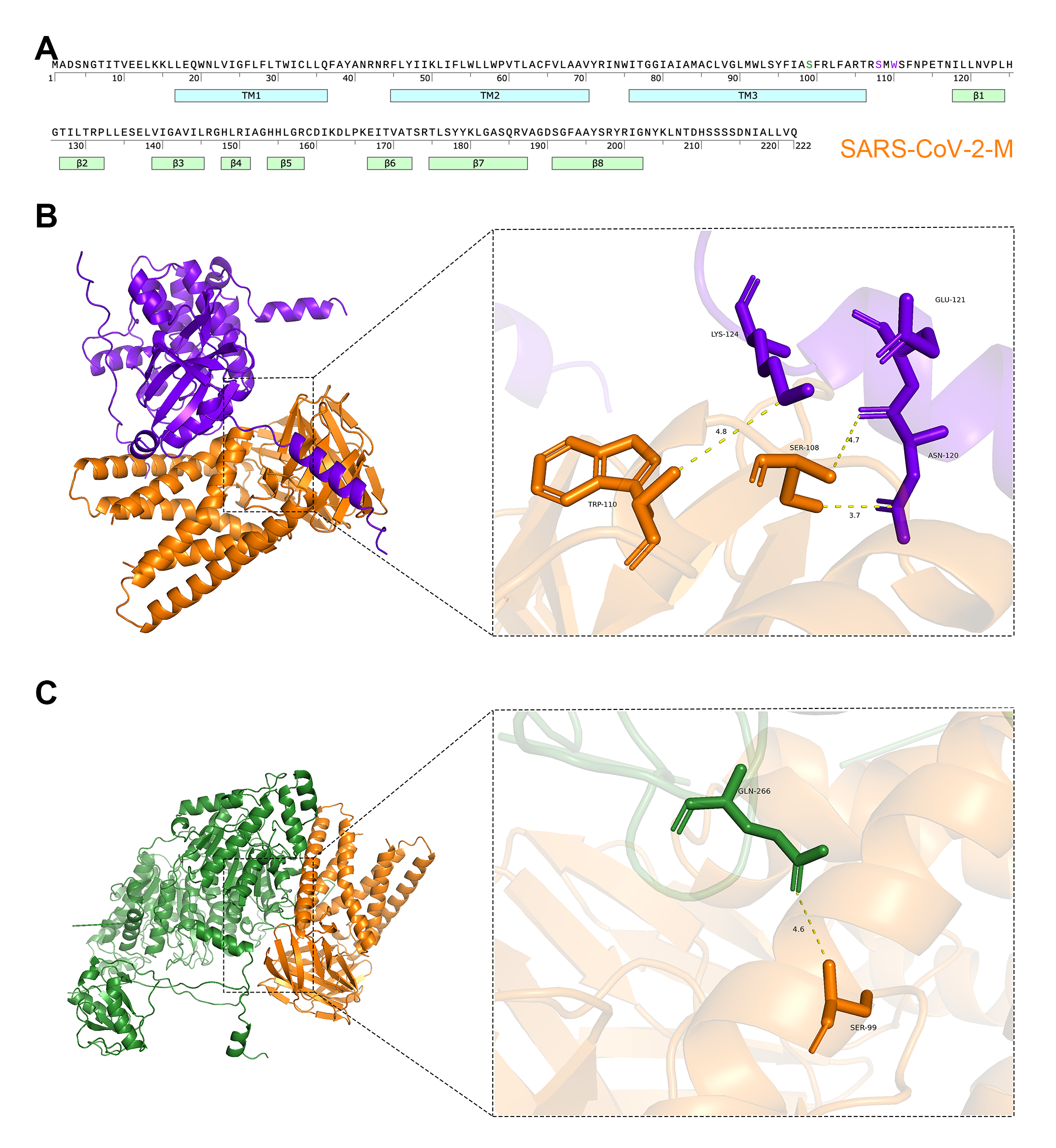

Supplement: S6 Fig — (A) The protein sequence and structural domains of SARS-CoV-2 M protein. (B-C) Illustrative models of mimic binding conformations between SARS-CoV-2 M protein (orange) and human AurA (purple-blue) (B) or HDAC6 (forest) (C). The mimic interaction sites are shown in an enlarged view and emphasized as model color in (B-C). (TIF) [file ppat.1013515.s006.tif]

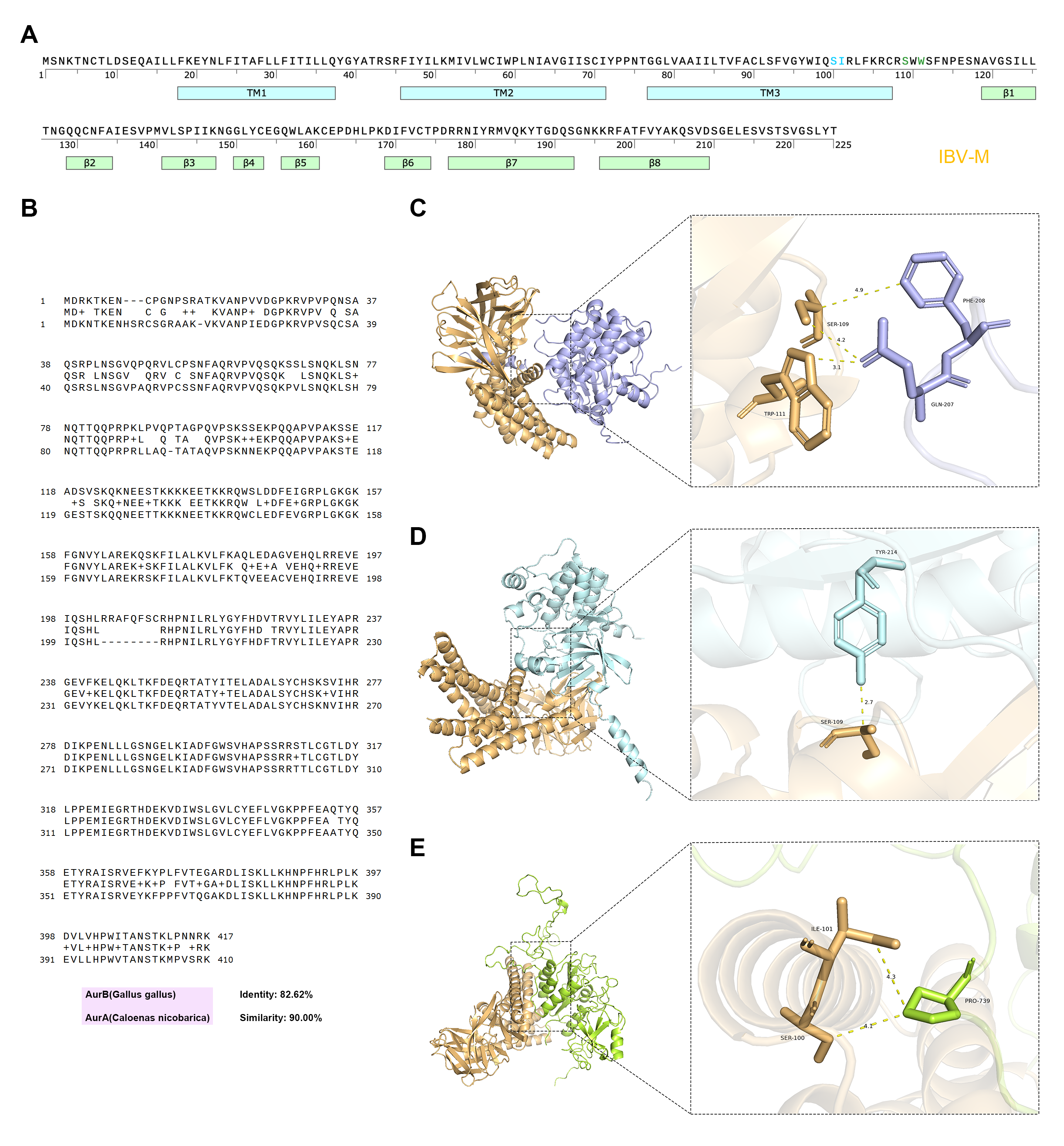

Supplement: S7 Fig — (A) The protein sequence and structural domains of IBV M protein. (B) Alignment between the protein sequence of chicken Aurora B (AurB) and Nicobar pigeon AurA, with identity and similarity percentages at 82.62% and 90.00%, respectively. (C-E) Illustrative models of mimic binding conformations between IBV M protein (light orange) and chicken AurB (light blue) (C), Nicobar pigeon AurA (pale cyan) (D) or HDAC6 (limon) (E). (TIF) [file ppat.1013515.s007.tif]
